# Stiffness and axial pain are associated with the progression of calcification in a mouse model of diffuse idiopathic skeletal hyperostosis

**DOI:** 10.1186/s13075-023-03053-3

**Published:** 2023-04-29

**Authors:** Dale E. Fournier, Matthew A. Veras, Courtney R. Brooks, Diana Quinonez, Magali Millecamps, Laura S. Stone, Cheryle A. Séguin

**Affiliations:** 1 Health and Rehabilitation Sciences (Physical Therapy), Faculty of Health Sciences, The University of Western Ontario, London, ON N6A 5B9 Canada; 2 Bone and Joint Institute, The University of Western Ontario, London, ON N6A 5C1 Canada; 3 Department of Physiology and Pharmacology, Schulich School of Medicine & Dentistry, The University of Western Ontario, London, ON N6A 5C1 Canada; 4 Faculty of Dentistry, McGill University, Montreal, QC H3A 1G1 Canada; 5 Alan Edwards Centre for Research on Pain, McGill University, Montreal, QC H3G 0G1 Canada; 6 Faculty of Medicine, Department of Anesthesiology, University of Minnesota, Minneapolis, MN 55455 USA

**Keywords:** Diffuse idiopathic skeletal hyperostosis (DISH), Spine mineralization, Pain, Spine stiffness, Behavioral measures of pain, Neuroplastic changes, Longitudinal analysis, Preclinical model, ENT1 knockout

## Abstract

**Background:**

Diffuse idiopathic skeletal hyperostosis (DISH) is characterized by progressive calcification of spinal tissues; however, the impact of calcification on pain and function is poorly understood. This study examined the association between progressive ectopic spine calcification in mice lacking equilibrative nucleoside transporter 1 (*ENT1*^*−/−*^), a preclinical model of DISH, and behavioral indicators of pain.

**Methods:**

A longitudinal study design was used to assess radiating pain, axial discomfort, and physical function in wild-type and *ENT1*^*−/−*^ mice at 2, 4, and 6 months. At endpoint, spinal cords were isolated for immunohistochemical analysis of astrocytes (GFAP), microglia (IBA1), and nociceptive innervation (CGRP).

**Results:**

Increased spine calcification in *ENT1*^*−/−*^ mice was associated with reductions in flexmaze exploration, vertical activity in an open field, and self-supporting behavior in tail suspension, suggesting flexion-induced discomfort or stiffness. Grip force during the axial stretch was also reduced in *ENT1*^*−/−*^ mice at 6 months of age. Increased CGRP immunoreactivity was detected in the spinal cords of female and male *ENT1*^*−/−*^ mice compared to wild-type. GFAP- and IBA1-immunoreactivity were increased in female *ENT1*^*−/−*^ mice compared to wild-type, suggesting an increase in nociceptive innervation.

**Conclusion:**

These data suggest that *ENT1*^*−/−*^ mice experience axial discomfort and/or stiffness and importantly that these features are detected during the early stages of spine calcification.

## Introduction

Diffuse idiopathic skeletal hyperostosis (DISH) is a non-inflammatory spondyloarthropathy characterized by the formation of ectopic mineral along the spine and within the annulus fibrosus of the intervertebral disc (IVD) [1, 2]. DISH is more frequently (but not exclusively) detected in males compared to females [3–5].

Overall, the clinical features of DISH are poorly understood. Multiple co-morbidities have been associated with DISH including obesity, dyslipidemia, hypertension, and type 2 diabetes [2]. The burden of pain in people living with DISH is unclear since studies have both suggested [6, 7] and refuted the presence of back pain [8, 9]. Instead, DISH is characterized clinically as increased spine stiffness, decreased spinal range of motion [6, 10], and postural changes (e.g., thoracic kyphosis) [11, 12]. In advanced stages, DISH is associated with dysphagia [13], dysphonia [14], airway obstruction [15], vertebral fracture [16], or spinal cord/nerve compression [17–19]. To date, the radiographic manifestations of DISH have not been directly correlated to symptoms. Further, since the etiology of DISH remains elusive, there are no disease-modifying or symptom-reducing treatments beyond surgical resection of mineralized tissue [20]. Instead, conservative management (e.g., physical therapy) is advocated with the goal of enhancing spine mobility; although, there is no convincing evidence showing its effectiveness to alter disease course [2, 21].

The lack of longitudinal studies evaluating the clinical features associated with DISH, including pain, is attributed to the fact that the current clinical radiographic criteria for diagnosis do not detect early disease [22]. As such, preclinical animal models are vital to better understand the pathobiology of DISH. Previous studies by our group reported that mice lacking equilibrative nucleoside transporter 1 (*ENT1*^*−/−*^) develop ectopic calcification of the paraspinal tissues with remarkable resemblance to DISH [23]. ENT1 is a ubiquitously expressed transmembrane protein that mediates the bi-directional transport of nucleosides such as adenosine across the plasma membrane [24]. *ENT1*^*−/−*^ mice show neurological changes resulting in potential disruption to circadian rhythms [25], ethanol preference [26, 27], and reduced anxiety [28]. Ectopic spinal calcification in *ENT1*^*−/−*^ mice is first detected in the cervical region at 2 months of age and progresses caudally; by 12 months of age, *ENT1*^*−/−*^ mice develop hind limb paralysis due to spinal cord compression [23]. By examining spinal tissues of mice lacking ENT1 [23] our group has confirmed similarities in the radiographic and histological features of mineralized tissues with those of human spines with DISH [29, 30]. Thus, the *ENT1*^*−/−*^ mouse serves as a useful preclinical model of DISH enabling longitudinal characterization of the onset and severity of symptoms related to ectopic calcification.

The current study presents a comprehensive longitudinal evaluation of physical function, mobility, and pain in the *ENT1*^*−/−*^ mouse model of DISH using behavioral and molecular testing modalities established in mouse models of IVD degeneration [31–34]. This analysis sought to examine the association between the progression of spine calcification and pain-related behavioral and molecular changes to better understand symptoms associated with the early stages of disease pathogenesis in DISH.

## Materials and methods

### Animals

Mice (*Mus musculus*) lacking ENT1 (B6.129X1-*Slc29a1*^*tm1Msg*^; referred to as *ENT1*^*−/−*^) were generated by deletion of exons two to four of the gene *Slc29a1* [27]. *ENT1*^*−/−*^ mice were backcrossed with C57BL/6NCrl mice (Charles River: Wilmington, MA) and heterozygous mice were bred to obtain wild-type and knock-out (*ENT1*^*−/−*^) littermates. Mice were housed in a facility with a controlled environment of temperature ranging from 22 to 25 °C, relative humidity ranging from 40 to 60%, 12 h light/dark cycles, and daily health assessments. Mice were housed in standard polycarbonate cages with two to four same-sex littermates, and ad libitum access to water and food (7013, Envigo: Madison, WI). Mice were euthanized at 6.5 months of age (196 ± 1.4 days) by intraperitoneal injection of 50 mg/kg of pentobarbital sodium (1EUF001, Bimeda^©^ Canada: Cambridge, CAN). All aspects of this study were conducted in accordance with the policies and guidelines set forth by the Canadian Council on Animal Care and were approved by the Animal Use Subcommittee of the University of Western Ontario (Protocol No. 2017–154).

### Characterization of physical function and behavioral indicators of pain

**Fig. 1:**
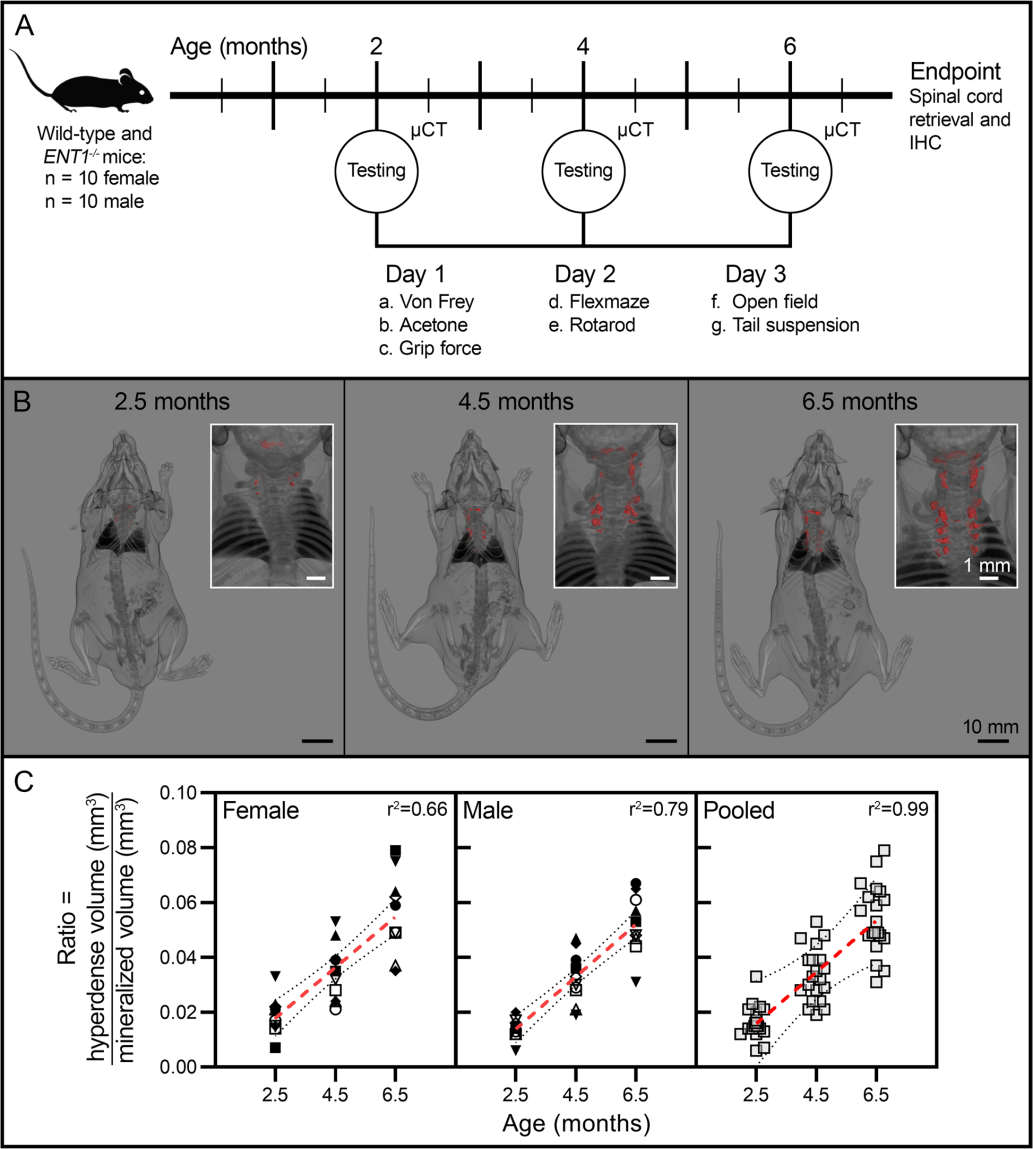
Experimental overview and microcomputed tomography (μCT)-based quantification of ectopic calcification in *ENT1*^*−/−*^ mice. **A** Wild-type (10 female, 10 male) and *ENT1*^*−/−*^mice (10 female, 10 male) were studied for behavioral indicators of pain and physical function at 2, 4, and 6 months of age. Behavioral assays were conducted over three testing days. µCT was performed to correlate the extent of ectopic calcification with behavior. **B** Three-dimensional renderings (anterior view) of the same representative male *ENT1*^*−/−*^ mouse at 2.5, 4.5, and 6.5 months of age. Inserts present magnified posterior view of the cervical-thoracic spine. Areas of hyperdense tissue, exceeding the radiodensity of vertebral cortical bone (≥ 1278 HU), are pseudocolored in red. **C** Ectopic calcification in *ENT1*^*−/−*^ mice quantified as the ratio of hyperdense tissue to vertebral bone (C1 to T12). Linear regression analysis with goodness of fit values presented as r^2^. Each animal is represented as a unique symbol. The slopes of the linear regressions were significantly non-zero with no differences between sexes (female, *y* = 0.0093*x* – 0.0007; male, *y* = 0.0096*x* – 0.0053). Data were pooled to analyze the overall relationship between ectopic calcification and age using the mean value for each time point (*y* = 0.0094*x* – 0.0030). Red lines represent the linear regression with dotted lines representing 95% confidence intervals

A total of 40 mice were studied (wild-type: 10 female, 10 male; *ENT1*^*−/−*^: 10 female, 10 male). Sample sizes were based on previous studies showing ectopic spine calcification in 100% of *ENT1*^*−/−*^ mice [23]. A longitudinal study design with repeated measures was employed to assess behavior at 2, 4, and 6 months of age (Fig. 1A). Time points were selected to assess the onset of ectopic calcification in *ENT1*^*−/−*^ mice [23, 35].

All mice were habituated to the behavioral testing facility 14 days prior to testing. Habituation to assays was performed seven days prior to testing. Testing was performed in the same sequence, by the same researcher, at the same time (12:00 to 18:00) over the span of three days (Fig. 1A). Each testing day included one hour of habituation to the testing room and was interspersed by one to two days without testing. Mice were returned to home cages after each assay for at least one hour to minimize interference between assays (except for von Frey/acetone and open field/tail suspension, which were tested consecutively).

### Behavioral assays

#### Flexmaze

The apparatus was based on previously published specifications [32]. Each mouse freely explored the maze for a single duration of 20 min. Videos were analyzed for the number of double gates passed over the 20-min test period by one observer blinded to genotype and sex.

#### Tail suspension

Spontaneous reaction to gravity-induced stretch was assessed in mice suspended by the base of their tail for 180 s [31]. The duration of time each mouse spent self-supporting (holding onto the tape, tail, or legs), rearing (reaching for limbs or tail), stretching (reaching for the floor), and immobile was analyzed using the ANY-maze™ video tracking system (Version 6.03, Stoelting Co.^©^: Wood Dale, IL) by two independent, blinded observers and averaged.

#### Open field activity

Voluntary locomotion was quantified for five minutes immediately pre- and post-tail suspension using open field boxes (0.4 × 0.4 × 0.3 m) equipped with tri-planar infrared sensors (VersaMax Legacy Open Field, Omnitech Electronics Inc.^©^: Columbus, OH). Measurements included total distance traveled and vertical activity (Fusion v5.6 r1159, Omnitech Electronics Inc.^©^: Columbus, OH).

#### Rotarod

Locomotor capacity was assessed with an accelerating rod (30 mm diameter; AccuRotor, Omnitech Electronics Inc.^©^: Columbus, OH). The rotation speed increased to 30 rpm over the course of 60 s and was maintained for an additional 240 s. The outcome measure was time to fall, detected by an infrared sensor. Animals that fell once within the first 30 s were immediately placed back onto the rod (within five seconds) and videos confirmed latency times.

#### Cold sensitivity

Cold sensitivity was assessed by quantifying the total time mice engaged in acetone-evoked behaviors (e.g., lifting, flicking, or licking the affected paw) after acetone was introduced to the plantar surface of the hind paw. Videos, 60 s in duration, were analyzed by an observer blinded to genotype and sex.

#### Mechanical sensitivity

Calibrated von Frey filaments (Stoelting Co.^©^: Wood Dale, IL) were applied to the plantar surface of each hind paw of mice for three seconds or until withdrawal, and the 50% withdrawal threshold was calculated [36]. The stimulus intensity ranged from 0.07 to 10.0 g, beginning at 1.4 g. The filaments used depended on the first filament to evoke a response, followed by five consecutive stimulations using the up-down method [36]. The scores from both hind paws were averaged.

#### Grip force

Grip force was measured as the maximum peak force produced (in grams) at the point of release by the forepaws during axial stretch, using a digital force gauge (Chatillon DFE Series, Ametek®: Berwyn, PA). For each mouse, peak grip force was measured three times at five minute intervals and the results were averaged.

### Microcomputed tomography (µCT)

*ENT1*^*−/−*^ mice were scanned following each time point of behavioral testing (2.5, 4.5, and 6.5 months of age) in order to correlate behavioral changes to radiographic features of spine calcification. µCT imaging was performed using a cone-beam imaging system (eXplore SpeCZT scanner, GE Healthcare Biosciences: London, CAN), as previously reported [23]. Mice were anesthetized for µCT scanning using two to three percent inhaled isoflurane (CA2L9100, Baxter: Mississauga, CAN) infused with oxygen at a flow rate of 1.0 mL/min. To maintain sedation, a nose cone apparatus was used to administer 1.75% inhaled isoflurane for 15 min while scanning was performed.

Quantitative analysis of vertebral cortical bone (three mice each time point) created a radiodensity range for mineralized tissue (289 to 1278 HU). The cervical-thoracic spine and sternocostal regions were manually segmented for analysis of hyperdense calcifications, defined as volumes exceeding the radiodensity of cortical bone (≥ 1278 HU) within these anatomical structures. Imaging data were analyzed using MicroView (Version 2.5.0–4118, Parallax Innovations Inc.: Ilderton, CAN) and VGStudio MAX (Version 2.0.4, Volume Graphics GmbH: Heidelberg, DEU).

### Immunohistochemistry

The cervical-thoracic spinal cord was collected at endpoint, fixed with 4% paraformaldehyde (12 to 18 h), and cryoprotected in 30% sucrose in 0.01 M phosphate-buffered saline for 24 h. The cervical enlargement of the spinal cord was dissected into thirds for each mouse. Tissues were embedded in O.C.T Compound™ (Tissue-Tek®: Alphen aan den Rijn, NLD), sectioned in the transverse plane at a thickness of 14 µm (CM3050 S, Leica Biosystems Nußloch GmbH: Nußloch, DEU), and thaw-mounted onto gelatin-coated slides.

Immunohistochemistry was performed as previously described [34, 37, 38] using primary antibodies directed against calcitonin gene-related peptide (CGRP, 1:750; BML-CA1137, Enzo Life Sciences: Farmingdale, NY), ionized calcium-binding adapter molecule 1 (IBA1, 1:1000; AB10341, Abcam: Cambridge, UK), and glial fibrillary acidic protein (GFAP, 1:500; G3893, Sigma-Aldrich: St. Louis, MI) with fluorescence-conjugated secondary antibodies diluted 1:500 in phosphate-buffered saline: donkey anti-sheep (A11015), donkey anti-rabbit (A21207), or donkey anti-mouse immunoglobulin G (A21202, Thermo Fisher Scientific: Waltham, MA). Following antibody incubations, sections were cover-slipped using Fluoroshield Mounting Medium with 4′,6-diamidino-2-phenylindole (ab104139, Abcam). Immunoglobulin G isotype controls for CGRP (1:750; 5–001-A, R&D systems: Minneapolis, MN), IBA1 (1:1000; 02–6102, Thermo Fisher Scientific), and GFAP (1:500; MA1-10,406, Thermo Fisher Scientific), as well as secondary-only controls, were run in parallel.

Tissue sections were imaged using a Leica Microsystems DMI6000B fluorescence microscope and DFC360FX camera with Leica Advanced Application Suite software (Version 2.7.0–9329, Leica Microsystems GmbH: Wetzlar, DEU). Micrographs were exported to ImageJ software and converted to 8-bit RGB stacks for analysis [39]. A baseline signal intensity threshold was generated for each antibody from the respective isotype and secondary-only controls. A region of interest was defined around each dorsal horn to analyze laminae one to four, based on greyscale density using brightfield images. The total area measured for analysis was standardized for each region of the cervical enlargement and the raw integrated density of immunoreactivity was averaged from three randomly selected sections from each region per animal.

### Statistical analyses

Statistical analyses were performed using GraphPad Prism (Version 8.0.1: San Diego, CA). Behavioral data from each mouse was analyzed as the experimental unit with repeated measures over time. A *P* value < 0.05 was considered significant.

The Shapiro–Wilk test was used to assess normality of data. µCT data were assessed using linear regression modeling. Behavioral data were analyzed using two-way-ANOVA with Sidak’s multiple comparisons for differences between genotypes and repeated measures. Mechanical sensitivity data were not normally distributed, so these data were analyzed using Mann–Whitney’s tests for differences between genotypes and Friedman’s tests with Dunn’s multiple comparisons for differences between repeated measures. Immunohistochemistry data were analyzed using unpaired *t*-tests for differences between genotypes and two-way-ANOVA with Sidak’s multiple comparisons to test differences between genotypes and regions.

## Results

### *ENT1*^*−/− *^mice show progressive ectopic calcification of the spine

Our previous studies demonstrated that loss of ENT1 function was associated with progressive ectopic calcification of paraspinal and IVD tissues in mice. In order to directly correlate radiographic features of spine calcification to behavioral changes, repeated µCT was used to quantify the extent of ectopic spine calcification in each *ENT1*^*−/−*^ mouse following behavioral testing (2.5, 4.5, and 6.5 months of age). This analysis also enabled us to assess the progression of ectopic calcification over time in both female and male mice. Hyperdense calcifications (exceeding the radiodensity of cortical bone) were first detected in *ENT1*^*−/−*^ mice at 2.5 months of age in the upper cervical spine (*n* = 20/20) and sternocostal articulations (*n* = 19/20) (Fig. 1B). Quantitative analysis of the cervical-thoracic spine and sternocostal tissues revealed a significant increase in the volume of hyperdense material with age in *ENT1*^*−/−*^ mice (female: Δ2.5 to 6.5 months = 4.4-fold ± 3.8 and male: Δ2.5 to 6.5 months = 4.7-fold ± 1.0). To account for age and sex-related differences in skeletal size, the extent of ectopic calcification was evaluated based on the ratio of hyperdense tissue relative to normal cortical bone within the same region of interest for each animal (Fig. 1C). Significant positive linear relationships were found between the ratio of hyperdense tissue to normal cortical bone and age in both female and male *ENT1*^*−/−*^ mice (Fig. 1C, *left and middle panels*), with the slope of the curves similar between female and male *ENT1*^*−/−*^ mice (*P* = 0.85). No significant differences were detected between female and male *ENT1*^*−/−*^ mice at any time point examined. As such, data from female and male mice were pooled to characterize the overall relationship between ectopic calcification and age in *ENT1*^*−/−*^ mice (Fig. 1C, *right panel*).

### Behavioral assessments of axial discomfort

#### Grip force assay

**Fig. 2:**
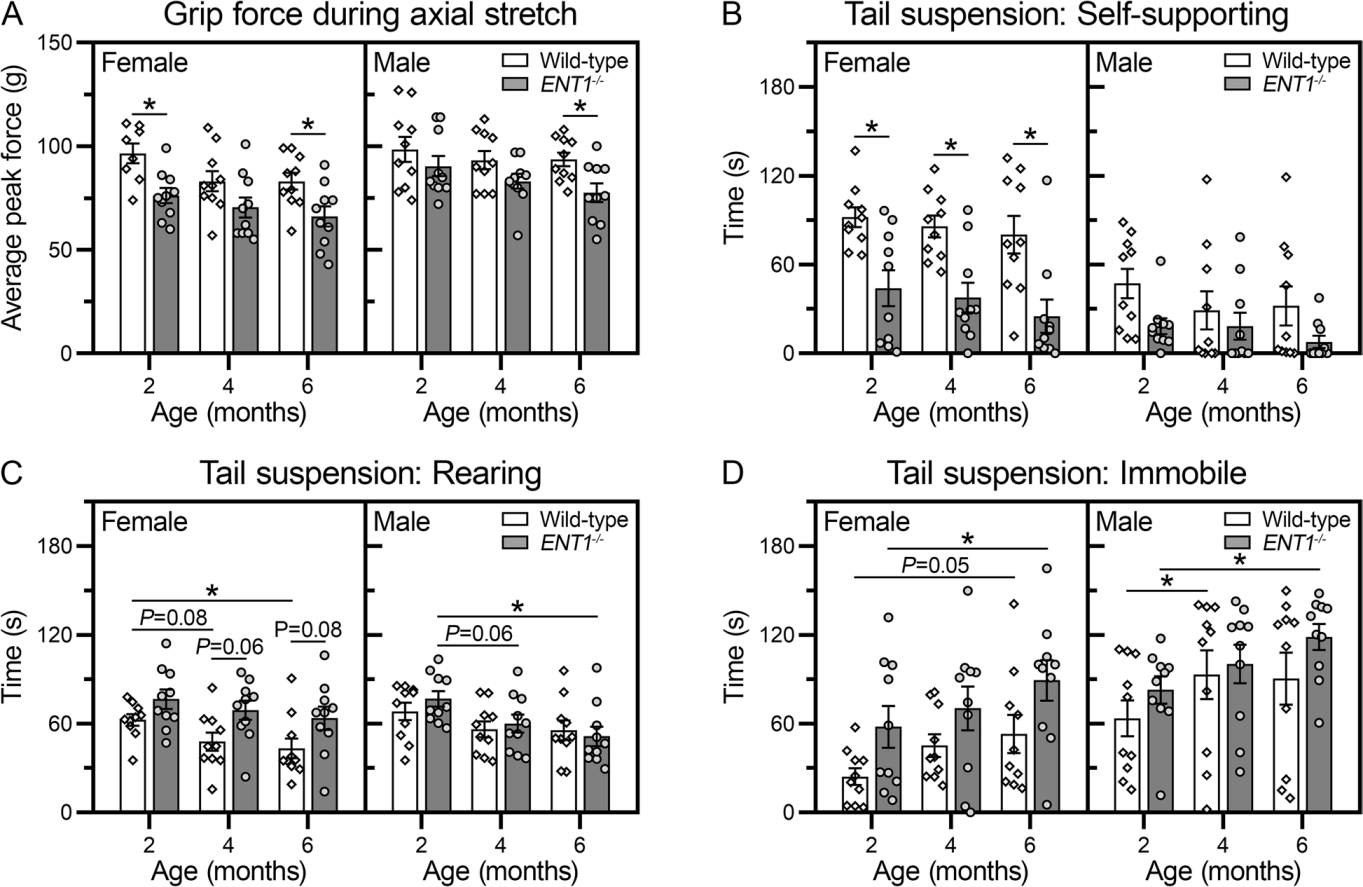
*ENT1*^*−/−*^ mice show reduced grip force without signs of axial stretch-induced discomfort in tail suspension. **A** In the grip force assay, the peak force at the point of release (measured in grams) was recorded during axial stretch. Data are stratified by sex, each animal is represented as a unique symbol, and data are presented as means ± SEM (*n* = 10 female, 10 male per genotype). Data from two female wild-type mice at 2 months of age are missing due to non-compliance with testing. **B**–**D** In the 180-second tail suspension assay, spontaneous behavioral responses to axial stretch are recorded: **B** self-supporting, **C** rearing, and **D** immobile. Data are stratified by sex, each animal is represented as a unique symbol, and data are presented as means ± SEM (*n* = 10 female, 10 male per genotype). *P < 0.05 by two-way-ANOVA or mixed-effects model with Sidak’s multiple comparisons test

A modified grip force assay was used to measure peak grip force during axial stretch [32–34]. Female *ENT1*^*−/−*^ mice demonstrated a significant reduction in grip force during stretch compared to wild-type mice at 2 and 6 months of age (Fig. 2A). Male *ENT1*^*−/−*^ mice showed reduced grip force compared to wild-type mice at 6 months of age.

#### Tail suspension assay

Previous studies using mouse models of lumbar IVD degeneration demonstrated that in tail suspension, mice experiencing stretch-induced axial pain spend more time self-supporting and rearing than healthy controls [31–34]. Overall, *ENT1*^*−/−*^ mice did not exhibit behaviors indicative of stretch-induced discomfort (Fig. 2B–D).

Female *ENT1*^*−/−*^ mice spent significantly less time self-supporting compared to age-matched wild-type mice (Fig. 2B). A similar but non-significant trend was observed in male *ENT1*^*−/−*^ mice. There were no differences with age in time spent self-supporting in either genotype or sex. Time spent rearing was not significantly different between wild-type and *ENT1*^*−/−*^ mice at any time point (Fig. 2C). Male *ENT1*^*−/−*^ mice showed a reduction in time spent rearing with age, with a significant reduction at 6 compared to 2 months of age. Time spent immobile was not significantly different between wild-type and *ENT1*^*−/−*^ mice at any time point (Fig. 2D). However, a significant increase in immobile time was observed with age in both female and male *ENT1*^*−/−*^ mice between 2 and 6 months of age (Fig. 2D).

### Behavioral assessments of physical function

#### Open field assay

**Fig. 3:**
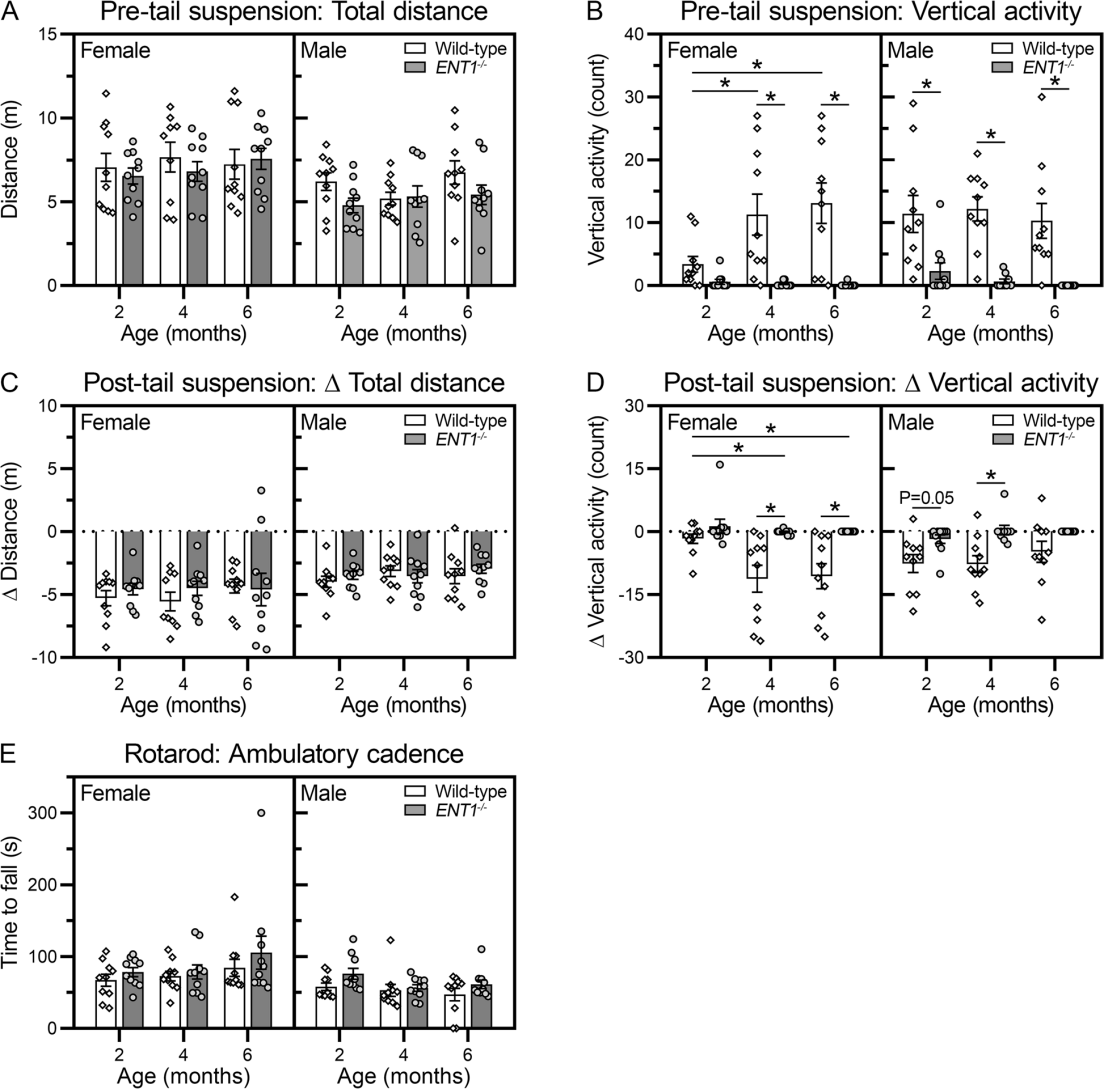
*ENT1*^*−/−*^ mice show reductions in voluntary rearing without overall changes in locomotor capacity. In the open field assay, baseline voluntary locomotion was assessed over 5 min. All data are stratified by sex, each animal is represented as a unique symbol, and data are presented as mean ± SEM (*n* = 10 female, 10 male per genotype). **A** Total distance traveled in meters. One female wild-type mouse at 4 months was excluded as an outlier, based on Grubb’s two-sided test (α = 0.01). **B** Vertical activity count measured by disruption of the *Z*-plane infrared sensor. One male *ENT1*^*−/−*^ mouse at 4 months was excluded as an outlier, based on Grubb’s two-sided test (α = 0.01). **C**, **D** The effect of axial stretch on voluntary physical function was measured over five minutes immediately following the tail suspension assay. A difference score was calculated as the change in activity pre- and post-tail suspension (Δ = post – pre). **E** In the rotarod assay, locomotor capacity was quantified by the time to fall for animals on an accelerating rotating rod. Data from two male wild-type mice at 6 months of age are missing due to non-compliance with testing. *P < 0.05 by two-way-ANOVA or mixed-effects model with Sidak’s multiple comparisons test

Voluntary locomotion was assessed in an open field. No significant differences were detected in the total distance traveled between wild-type and *ENT1*^*−/−*^ mice at any time point (Fig. 3A). Similarly, no differences were detected in complementary voluntary locomotion metrics, including total movement or rest time. Conversely, a marked reduction in vertical activity (rearing on hind limbs) was observed in *ENT1*^*−/−*^ mice compared to wild-type at all time points assessed (Fig. 3B).

We next evaluated the effect of gravitational axial stretch on voluntary locomotion by comparing activity in an open field pre- and post-tail suspension. Tail suspension induced a reduction in voluntary activity (total distance traveled) in all groups assessed; however, no differences were detected between wild-type and *ENT1*^*−/−*^ mice (Fig. 3C). Tail suspension induced a reduction in vertical activity in wild-type mice. No vertical activity change from baseline was detected in *ENT1*^*−/−*^ mice since they demonstrated minimal vertical activity prior to tail suspension (Fig. 3D).

#### Rotarod assay

We investigated if *ENT1*^*−/−*^ mice demonstrated altered locomotor capacity using an accelerating rotarod assay. No significant difference was detected in *ENT1*^*−/−*^ mice compared to wild-type at any of the time points examined in either female or male mice (Fig. 3E). Moreover, no change in performance was detected with age for either genotype or sex.

#### Flexmaze assay

**Fig. 4:**
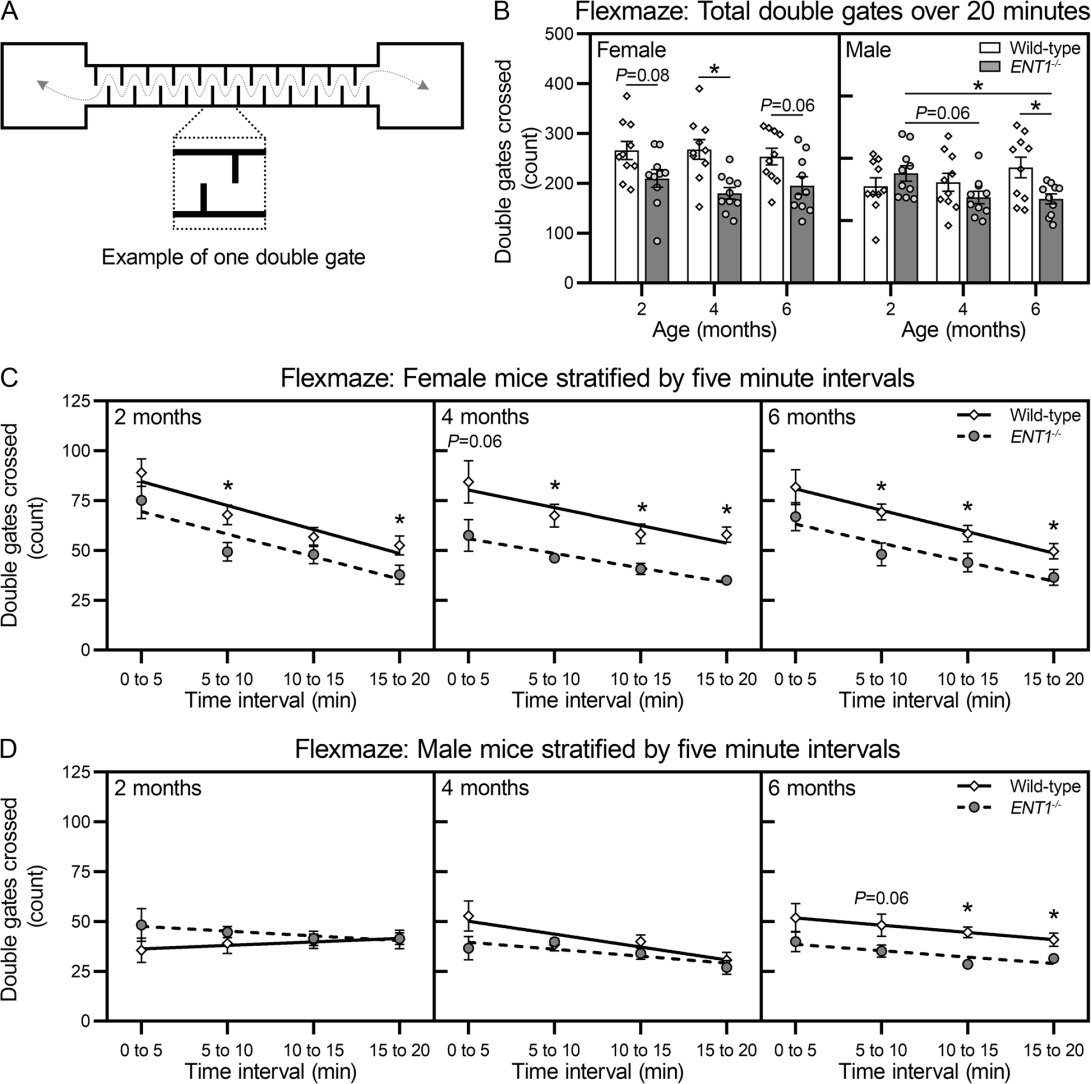
*ENT1*^*−/−*^ mice show reduced physical function in the flexmaze assay. **A**–**C** In the flexmaze assay, ambulation is measured as the number of double gates crossed per minute during the 20-minute test. Data are stratified by sex, each animal is represented as a unique symbol, and data are presented as means ± SEM (*n* = 10 female, 10 male per genotype). **A** The total number of double gates crossed during the full duration of the test for both sexes. *P < 0.05 by two-way-ANOVA with Sidak’s multiple comparisons test. **B**, **C** The 20 min test was segmented into five-minute intervals (0 to 5, 5 to 10, 10 to 15, and 15 to 20 min). Data are expressed as means ± SEM and are fitted with linear regressions. *P < 0.05 by unpaired, two-tailed *t*-test for differences between sex-matched wild-type and *ENT1*^*−/−*^ mice at each interval

In the flexmaze assay, mice are forced to undergo lateral spine flexion to pass through the staggered gates of the maze (Fig. 4A) [32]. When the average number of double gates passed over the total assay duration was assessed, female *ENT1*^*−/−*^ mice showed less activity than age-matched wild-type mice, with a significant reduction at 4 months of age (Fig. 4B). Male *ENT1*^*−/−*^ mice demonstrated reduced activity compared to age-matched wild-type mice at 6 months of age and showed a significant decrease in flexmaze activity with age (between 2 and 6 months).

Intriguing differences were noted in exploratory behavior in the flexmaze when assessed as a function of time (Fig. 4C, D). Activity of female mice decreased during the assay in both wild-type and *ENT1*^*−/−*^ mice at all time points (Fig. 4C). At 2 months of age, female *ENT1*^*−/−*^ mice showed reduced activity compared to wild-type mice during the 5 to 10 and 15 to 20 min intervals. At 4 and 6 months of age, female *ENT1*^*−/−*^ mice showed reduced activity during the latter 15 min of the assay compared to wild-type mice. Activity of male mice in the flexmaze decreased over the duration of the assay in wild-type at 4 months of age and *ENT1*^*−/−*^ mice at 4 and 6 months of age (Fig. 4D). No differences in activity were detected between male wild-type and *ENT1*^*−/−*^ mice at 2 or 4 months of age. At 6 months of age, male *ENT1*^*−/−*^ mice showed reduced activity compared to wild-type mice during the final 10 min of the assay.

### Behavioral assessments of sensitivity to cutaneous sensory stimuli

**Fig. 5:**
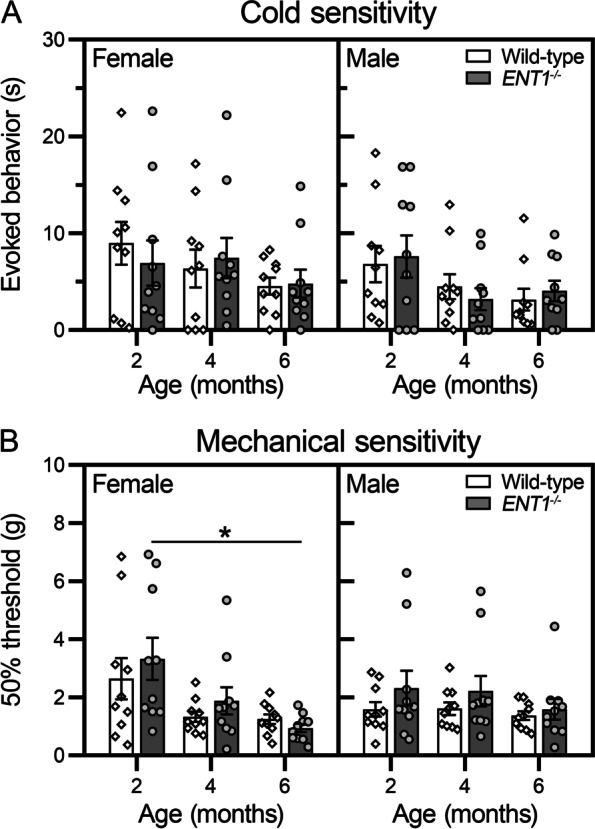
*ENT1*^*−/−*^ mice show no difference in hind limb sensitivity to cold or mechanical stimulation. All data are stratified by sex, each animal is represented as a unique symbol, and data are presented as means ± SEM (*n* = 10 female, 10 male per genotype). **A** To assess cold sensitivity, acetone was introduced to the plantar surface of the hind paw and the amount of time mice spent in acetone-evoked behaviors was recorded (e.g., lifting, flicking, or licking the affected paw). *P < 0.05 by two-way-ANOVA or mixed-effects model with Sidak’s multiple comparisons test. **B** In the von Frey assay, mechanical sensitivity is measured by the response to the manual application of calibrated filaments to the plantar surface of the hind paw using the Chaplan up-down method. No differences between left and right hind paws were detected using Mann–Whitney’s test, as such data are presented as averaged from both paws. *P < 0.05 by Mann–Whitney tests for differences between genotype and Friedman’s with Dunn’s multiple comparisons test for differences between time points

We assessed cutaneous sensitivity in the hind limbs, which in mouse models of lumbar IVD degeneration is indicative of radiating pain [31–34]. No significant differences were detected in hind paw sensitivity to cold between wild-type and *ENT1*^*−/−*^ mice for either female or male mice at any time point (Fig. 5A). Similarly, no significant differences were detected in hind limb mechanical sensitivity between wild-type and *ENT1*^*−/−*^ mice at any time point, in either female or male mice (Fig. 5B).

### *ENT1*^*−/−*^ mice show neuroplastic changes in the cervical spinal cord

**Fig. 6:**
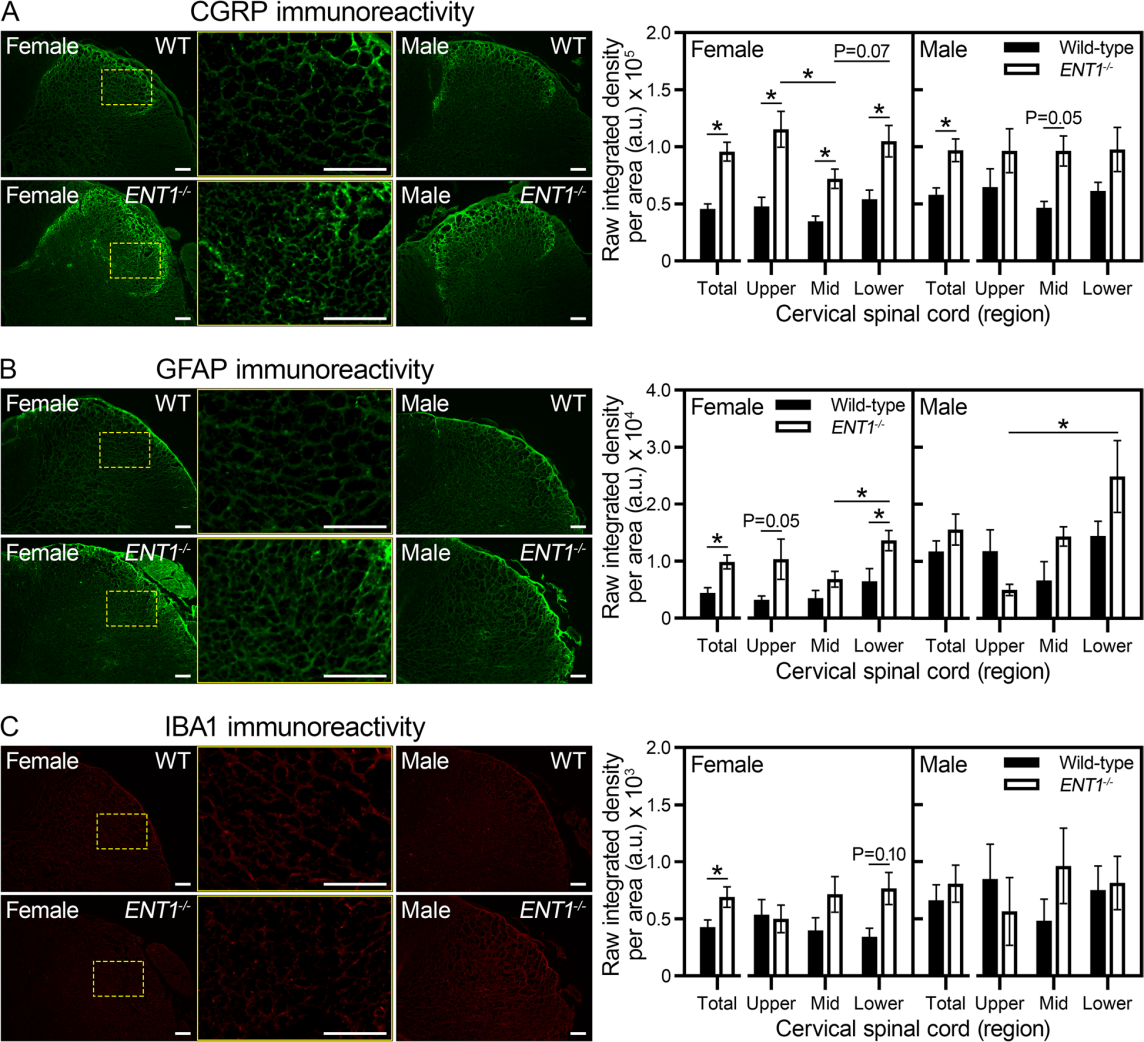
*ENT1*^*−/−*^ mice show neuroplastic changes in the cervical enlargement of the spinal cord at 6.5 months of age. **A**–**C** Representative images of immunofluorescent detection and quantification within the dorsal horn of the cervical spinal cord in wild-type and *ENT1*^*−/−*^ mice. No differences were detected between left and right dorsal horns using Mann–Whitney’s test, so data were pooled (*n* = three to four female, three male per genotype). To account for anatomical and size differences in the region of interest, the upper, mid, and lower regions of the cervical enlargement were analyzed independently. A region of interest was defined around each dorsal horn to analyze laminae one to four, based on greyscale density using brightfield images. The area was standardized for each region of the cervical enlargement and the raw integrated density of immunoreactivity was averaged from up to three randomly selected sections from each region per animal. Immunoreactivity was measured for **A** calcitonin gene-related peptide (CGRP), **B** glial fibrillary acidic protein (GFAP), and **C** ionized calcium-binding adapter molecule 1 (IBA1). Data are plotted as mean ± SEM. *P < 0.05 by Mann–Whitney test for genotype differences and Kruskal–Wallis with Dunn’s multiple comparisons test for differences between cervical regions

To further investigate the association between ectopic spine calcification and indicators of pain in *ENT1*^*−/−*^ mice, we assessed molecular markers of neuroplastic changes. We focused our analysis on the cervical enlargement of the spinal cord of mice at endpoint of the longitudinal study (6.5 months of age) since in *ENT1*^*−/−*^ mice the cervical region is the most severely affected by ectopic calcifications, which have been present for several months. Pain can be modulated by the neurotransmitter CGRP and increased expression is associated with nerve hyperexcitability and sensitization [40]. In both female and male mice, CGRP immunoreactivity in the dorsal horn of the cervical spinal cord was elevated in *ENT1*^*−/−*^ mice relative to wild-type (Fig. 6A). No differences were detected in CGRP immunoreactivity between female and male *ENT1*^*−/−*^ mice.

Increased levels of GFAP, a marker of astrocytes [41], and IBA1, a marker of microglia [42], are associated with increased hyperalgesia. Both GFAP and IBA1 immunoreactivity within the dorsal horn of the cervical spinal cord were significantly increased in female *ENT1*^*−/−*^ mice compared to wild-type (Fig. 6B, C). No significant differences were detected in either GFAP or IBA1 immunoreactivity in the cervical spinal cord of male *ENT1*^*−/−*^ mice compared to wild-type. Moreover, no differences were detected in total GFAP or IBA1 immunoreactivity between female and male *ENT1*^*−/−*^ mice.

## Discussion

The clinical features of DISH are poorly understood, generalized as spine stiffness, with or without back pain, based on limited cases reporting on advanced disease with extensive spine calcification [6, 10]. As such, the impact of physical dysfunction and pain for people living with DISH along the continuum of disease progression is poorly characterized. This investigation reports on behavioral indicators of physical function and pain in a preclinical mouse model of DISH. We demonstrate that, compared to age- and sex-matched wild-type mice, *ENT1*^*−/−*^ mice show alterations in physical function as reflected by decreased grip force, vertical activity in an open field, and activity in the flexmaze. Importantly, particularly in female mice, these changes are detected with the onset of ectopic spine calcification. Furthermore, the behavioral changes in *ENT1*^*−/−*^ mice at 6.5 months of age were associated with evidence of neuroplastic changes in the cervical spinal cord including increased immunolocalization of CGRP, GFAP, and IBA1. Taken together, these findings suggest that *ENT1*^*−/−*^ mice have alterations in physical function, discomfort/stiffness, and pain associated with the onset and progression of ectopic spine calcification.

In humans, ectopic spine calcification associated with DISH occurs over an extended period of time, typically initiating decades prior to diagnosis [43]. It is unknown when along this continuum that symptoms impact physical function or quality of life. The *ENT1*^*−/−*^ mouse recapitulates key aspects of DISH pathogenesis, including the temporal pattern of progression and spinal tissues affected [23]. In contrast to DISH however, which has a reported increased incidence in males compared to females [2], ectopic calcification of spinal tissues is detected to a similar extent in both female and male *ENT1*^*−/−*^ mice. In the current study, we show that behavioral signs of impaired physical function and discomfort/stiffness were detected in *ENT1*^*−/−*^ mice at 2 months of age, when ectopic calcification is first detected [23], and increased over time with the progression of ectopic calcification. These findings raise the possibility that the onset of ectopic calcification of spinal tissues in DISH may likewise impair physical function and cause symptoms.

Similar to mouse models of lumbar IVD degeneration [32, 44], *ENT1*^*−/−*^ mice showed reduced grip strength during axial stretch and reduced activity in the flexmaze assay, which requires lateral spine flexion. However, in contrast to mouse models of IVD degeneration [32, 44], *ENT1*^*−/−*^ mice showed reduced self-supporting in tail suspension compared to age-matched wild-type mice. While it is not clear if *ENT1*^*−/−*^ mice experience stretch-induced discomfort similar to mice with IVD degeneration, significant differences in behaviors detected in both female and male *ENT1*^*−/−*^ mice suggest that the change in spine kyphosis [23] and/or biomechanical stiffening of the spine [45] in the *ENT1*^*−/−*^ mice result in discomfort and/or stiffness that decreases overall spine mobility. Interestingly, similar traits have been reported in people with DISH, including change in kyphosis, reduced lateral flexion of the thoracic spine [6, 46], and reduced grip strength, the latter is used in the clinic as a measure of chronic pain and/or general upper limb function [6, 12, 47].

The similarity between the performance of wild-type and *ENT1*^*−/−*^ mice in the rotarod and open field assays suggests a lack of general functional impairment associated with loss of ENT1 or spine calcification. Moreover, *ENT1*^*−/−*^ mice did not show evidence of mechanical or cold hypersensitivity in the hind paw, suggesting the absence of radiating pain. This is likely attributed to ectopic calcifications limited to the cervical and upper thoracic spine between 2 and 6 months of age. However, the reduction in flexmaze activity and almost complete absence of vertical activity (rearing) by *ENT1*^*−/−*^ mice in open field suggests physical dysfunction and/or avoidance of discomfort-inducing activities. These behavioral measures are supported by molecular evidence of neuroplastic changes in *ENT1*^*−/−*^ mice. Increased levels of CGRP in the cervical spinal cord of *ENT1*^*−/−*^ mice confirm neuroplasticity in primary nociceptive neurons. Furthermore, elevated levels of GFAP and IBA1 detected in female *ENT1*^*−/−*^ mice suggest alterations in the sensory nervous system. These changes are consistent with mouse models of IVD degeneration that show axial pain [48] and suggest activation of pain signaling pathways in *ENT1*^*−/−*^ mice.

In the current study, behavioral and neural changes were more robust in female than male *ENT1*^*−/−*^ mice despite similar levels of ectopic spine calcification. These findings are consistent with previous studies in rodent models and humans establishing sex-specific differences in pain perception [49]. Female *ENT1*^*−/−*^ mice showed significant differences in the grip force, tail suspension, and flexmaze assays at earlier stages of spine calcification than did male *ENT1*^*−/−*^ mice. Since ectopic calcification was not different between female and male mice at any time point, these findings suggest that female *ENT1*^*−/−*^ mice are more susceptible to discomfort or pain. While it is unclear what mechanism underlies the sex-specific differences in behavioral signs of pain, estrogen-modulated cyclooxygenase (COX)-2 activation has been proposed as a potential mediator [49] and could be examined in *ENT1*^*−/−*^ mice in subsequent studies.

A strength of the current study is the longitudinal evaluation of behavioral indicators of physical function and discomfort with the initiation of spine calcification. Overall, significant differences between wild-type and *ENT1*^*−/−*^ mice, indicative of stiffness and/or discomfort, increased as spine calcification progressed. This suggests that the presence of small or focal ectopic calcifications may be insufficient to produce definitive symptoms. Instead, ectopic calcification may have to reach a certain extent and/or affect specific anatomy before symptoms can be detected in *ENT1*^*−/−*^ mice. A similar threshold effect may also underlie the variability in pain reported in humans with DISH and underscores the importance of treatment strategies aimed at delaying the progression of ectopic calcification and maintaining physical function. In fact, a small study of exercise therapy to improve spinal range of motion for people living with DISH reported improvements in physical measures and reductions in self-reported pain [50].

There are several limitations that should be acknowledged in the interpretation of our findings. First, the assessment of pain in animal models using behavioral assays requires careful experimental design as assays are sensitive to different types of pain and no single assay conclusively demonstrates that an animal is experiencing pain. To address this limitation, we included multiple complementary assays that together inform on behavioral indicators of pain, as well as objective measurement of neuroplastic changes within the central nervous system. Second, the *ENT1*^*−/−*^ mouse model differs from DISH in that the appendicular skeleton is not affected by ectopic calcification in the mouse. It is likely that calcification within appendicular joints would affect physical function and cause pain/discomfort similar to the spinal changes in *ENT1*^*−/−*^ mice, leading to a different pain profile in people with DISH. Finally, further studies are required to differentiate symptoms of pain and physical dysfunction in *ENT1*^*−/−*^ mice, for example by pharmacological targeting of inflammatory and nociceptive pain pathways at specific stages of spine calcification or by specifically investigating neuroplastic changes in mice at earlier time points associated with the onset of spine calcification and behavioral changes.

## Conclusions

Taken together, our results provide the first evidence that impaired physical function, axial stiffness and/or discomfort are features associated with spine calcification in *ENT1*^*−/−*^ mice. Importantly, behavioral changes were noted early in disease pathogenesis and increased as the severity of ectopic spine calcification progressed. These findings underscore that pain should be evaluated in people living with DISH throughout the progression of spine calcification, with emphasis on early-stage disease. To date, the most comprehensive case–control study concluded that chronic back pain, specifically in the cervical and thoracic regions of the spine, and upper extremity pain are significant clinical features of DISH [6]. Although mechanisms underlying discomfort were not addressed in the current investigation, our results provide the foundation for the application of future targeted treatments to alleviate symptoms of and/or modify the progression of spine calcification in a preclinical model of DISH.

## Data Availability

All datasets generated or analyzed during this study are included in this published article.

## Abbreviations

CGRP: Calcitonin gene-related peptide
DISH: Diffuse idiopathic skeletal hyperostosis
ENT1: Equilibrative nucleoside transporter 1
GFAP: Glial fibrillary acidic protein
IBA1: Ionized calcium-binding adapter molecule 1
IVD: Intervertebral disc
µCT: Microcomputed tomography
